# Under-reporting of adverse drug reactions: Surveillance system evaluation in Ho Municipality of the Volta Region, Ghana

**DOI:** 10.1371/journal.pone.0291482

**Published:** 2023-09-12

**Authors:** Richard Osei Buabeng, Paul Dsane-Aidoo, Yaw K. Asamoah, Delia Akosua Bandoh, Yvonne Adu Boahen, George Tsey Sabblah, Delese Mimi Darko, Charles Noora Lwanga, Donne Kofi Ameme, Ernest Kenu

**Affiliations:** 1 Ghana Field Epidemiology and Laboratory Training Programme, Department of Epidemiology and Disease Control, School of Public Health, College of Health Sciences, University of Ghana, Accra, Ghana; 2 World Health Organization, Country Office, Accra, Ghana; 3 Food and Drugs Authority, Accra, Ghana; University of Kwazulu-Natal, SOUTH AFRICA

## Abstract

**Background:**

Adverse Drug Reactions (ADRs) can occur with all medicines even after successful extensive clinical trials. ADRs result in more than 10% of hospital admissions worldwide. In Ghana, there has been an increase of 13 to 126 ADR reports per million population from 2012 to 2018. ADR Surveillance System (ADRSS) also known as pharmacovigilance has been put in place by the Ghana Food and Drugs Authority (FDA) to collect and manage suspected ADR reports and communicate safety issues to healthcare professionals and the general public. The ADRSS in Ho Municipality was evaluated to assess the extent of reporting of ADRs and the system’s attributes; determine its usefulness, and assess if the ADRSS is achieving its objectives.

**Methods:**

We evaluated the ADRSS of the Ho Municipality from January 2015 to December 2019. Quantitative data were collected through interviews and review of records. We adapted the updated CDC guidelines to develop interview guides and a checklist for data collection. Attributes reviewed included simplicity, data quality, acceptability, representativeness, timeliness, sensitivity, predictive value positive and stability.

**Results:**

We found a total of 1,237 suspected ADR during the period, of which only 36 (3%) were reported by healthcare professionals in the Ho Municipality to the National Pharmacovigilance Centre (NPC). Only 43.9% of health staff interviewed were familiar with the ADRSS and its reporting channel. Staff who could mention at least one objective of the ADRSS were 34.2%, and 12.2% knew the timelines for reporting ADR. Reports took a median time of 41 (IQR = 25, 81) days from reporter to NPC. Reports sent on time constituted 37.5%. Fully completed case forms constituted 77.1% and the predictive value positive (PVP) was 20%. About 53% of ADRs were reported for female patients. Up to 88.9% of ADRs were classified as drug related. Anti-tuberculosis agents and other antibiotics constituted (40.6%) and (18.8%) of all reports. The ADRSS was not integrated into the disease surveillance and response system of Ghana’s Health Service and so was not flexible to changes. A dedicated ADR surveillance officer in regions helped with the system’s stability. Data from Ghana feeds into a WHO database for global decision making.

**Conclusions:**

There was under-reporting of ADRs in the Ho Municipality from January 2015 to December 2019. The ADR surveillance system was simple, stable, acceptable, representative, had a strong PVP but was not flexible or timely. The ADRSS was found useful and partially met its objectives.

## Introduction

All medications have the potential to cause adverse reactions, even if they have been granted marketing authorization. Adverse Drug Reactions (ADR) are thought to be responsible for more than 10% of hospital admissions globally [1–3]. These admissions place a high financial burden on healthcare and some countries may spend up to 30.1 billion US dollars annually in the management of ADRs [4–6]. An assessment of 43 observational studies found that the incidence of ADR-related hospitalization in developed and developing countries was similar [7]. However, the expense of ADR-related hospitalizations is proportionately greater in developing countries, owing in part to a high disease burden, malnutrition, and resource-constrained healthcare systems [8].

While national medicines regulatory agencies (NMRAs) and other organizations perform ADR surveillance as part of their post-market authorization activities to ensure the safety of these medicines, the major obstacle to a globally viable ADR surveillance system (ADRSS) is under-reporting of ADRs [9, 10]. The median under-reporting rate for ADRs to spontaneous (passive) reporting systems was estimated to be 94% (IQR = 82, 98) in an analysis of 37 studies across 12 countries [11]. When the focus is on Africa, the situation is grave in terms of under-reporting [12]. In the Greater Accra Region of Ghana, research on under-reporting of ADRs discovered that only 21% of physicians reported ADRs in 2011, although 59.5% had encountered a patient with an ADR [13]. A similar study among community pharmacists reported that only 16% of those who had seen a patient with a suspected ADR in 2015 reported it [14].

In Ghana, the Food Drugs Authority (FDA) acts as the National Pharmacovigilance Centre (NPC) and is responsible for ensuring the safety of pharmaceuticals on the market, collecting and managing ADR reports, as well as communicating safety concerns to healthcare professionals, patients, and other stakeholders. The FDA’s newsletter, *DrugLens*, suggested that the ADR reporting is on the increase in recent years [15]. The number of reports received in 2018 was the highest in a single year and represented a sharp increase of 13 per million population in 2012 to 126 per million population. This, however, is below the 200 per million inhabitants frequently used as the standard for a well-performing system [16–18].

The objectives of the ADR Surveillance System are to assess and monitor risks related to the utilization of medical products in humans; to implement measures to reduce such risks; to promote the proper and safe use of these products and to generate signals to reported information on a possible causal relationship between an adverse event and a drug.

The Ministry of Health, Ghana, requires that surveillance systems be evaluated on an ongoing basis [19]. However, the ADRSS in the Ho Municipality, Volta Region–Ghana, had never been evaluated since its establishment in 2005. We aimed to study the reporting of ADRs, as well as evaluate the ADRSS to assess the system’s attributes, determine its usefulness, and assess if the ADRSS is achieving its objectives.

## Methods

### Evaluation design

We evaluated the ADRSS of the Ho Municipality from January 2015 to December 2019. Quantitative data were collected through interviews and review of records. There are a few validated tools for assessing pharmacovigilance including the World Health Organization Global Benchmarking Tool (WHO GBT), WHO Pharmacovigilance Indicators, and CDC Updated Guidelines for Evaluating Public Health Surveillance Systems [20, 21] collection [22]. However, we used the CDC Updated Guidelines for Evaluating Public Health Surveillance Systems to develop interview guides and a checklist for data collection [22]. This tool evaluates public health surveillance systems, focusing on how effectively the system performs in terms of meeting its purpose and objectives. This evaluation tool was chosen above the others because its purpose is directly related to the goal of this study—measuring the usefulness of the system and establishing if the ADRSS is fulfilling its objectives. Attributes reviewed included simplicity, data quality, acceptability, representativeness, timeliness, sensitivity, predictive value positive and stability.

### Study site and population

Ho Municipality is one of the 25 administrative districts in the Volta Region of Ghana and had a projected population size of 218,948 in 2019 [23]. The projected population size for Ho Municipal in 2015 was 199,844; in 2016 was 204,323; in 2017 was 209,212; and in 2018 was 213,960. This projection is based on the population of the Municipality which stood at 177,281 during the 2010 census with a growth rate of 2.5% [23, 24]. It is located in the middle zone of the Region and bordered in the north-west by Ho West District, east and South-east by Adaklu-Anyigbe district, and North-east by The Republic of Togo. The Municipality constitutes five sub-districts namely Hopketa, Norvisi, Dutasor, Sokode/Akrofu and Ho Central. The principal care provider at all levels of the health system is the Ghana Health Service (GHS) with the private sector, and Christian Health Association of Ghana (CHAG) complementing its activities. There were 56 health facilities in the Municipalities– 37 Community-based Health Planning and Services (CHPS), 8 Health Centres, 1 Polyclinic, 2 hospitals (1 municipal and 1 regional), 2 CHAG, 1 Quasi-Government and 5 Private Hospitals.

### Data collection

The Municipality’s health facilities had been classified into priority groups as high, medium, and low based on out-patient department (OPD) attendance by the Municipal Health Directorate. Hospitals (district and regional hospitals and above) were in the high group, Health Centres in the medium group and CHPS in the low group. Due to resource constraint, four facilities were randomly selected from each priority group. The names of all health facilities were written on pieces of papers and placed in a box as per priority group. The pieces of papers were picked without replacement until the desired sample size was achieved. Two community pharmacies were selected through convenience sampling. Stakeholders were engaged from health facilities, districts the district, regional and national levels. In each facility, we randomly selected a department and interviewed available staff at work. We included personnel who had been tasked by the facility to report ADRs (Institutional Contact Persons—ICP) in the in-depth interviews. Records review included data from annual, quarterly and monthly reports by the district, regional and national levels. The ADR reporting forms known as the ‘Blue Forms’ that came from Ho Municipality for the past 5 years were collected and reviewed. The number of reports, the type of ADR and reporting facility, the sex of those who had ADR and the reporter profession were analyzed.

#### Assessing under-reporting of ADRs

Annual reports and registers of the Municipal Health Directorate (MHD) and the facilities visited were reviewed to identify any missing ADRs. An ADR was considered missing if it was detected in a health facility or MHD but not reported to the NPC within the calendar year These were suspected ADR reports captured by a healthcare professional for onward submission to the NPC for causality assessment.

For the purpose of this study, a suspected ADR is considered reported when the ADR form reaches the NPC. A suspected ADR that was documented by the healthcare provider or patient and sent to the MHD but did not end up in NPC is not considered reported. Thus, ‘reported’ is from the perspective of the NPC and not the HCP. Causality assessments are not done for reports that end up at the MHD and do not contribute to signal detection. The ADR report should have had details of the patient, the reaction and the suspected product. The gap difference between missing ADRs and those reported to the NPC was computed. Reports that were sent to the NPC were compared to the WHO target.

Causality assessments for the reports were done by an independent group of experts at the national level using WHO-UMC System for Standardized Case Causality Assessment Scale [29]. The WHO-UMC causality assessment method considers clinical-pharmacological aspects and quality of documentation in assessing case reports for adverse drug reactions. It prioritizes the detection of unknown and unexpected reactions and uses four criteria to categorize causal association into six categories, with additional categories for unclassified or inaccessible reports.

#### Assessing whether the ADR surveillance system meets its objectives

Evidence of charts derived from the reports received was assessed to confirm ongoing trend monitoring. Also, to confirm whether the reports sent through the ADRSS from the MHD contributed to signal generation–reported information on a possible causal relationship between an adverse event and a drug; the relationship normally is unknown or incompletely documented previously, at the national level. Minutes from expert committee meetings and feedback letters were evaluated to determine whether causality assessments were done for ADRs reported from Ho Municipality. Since the ADRSS runs from the Health facility level to the Regional and National level respectively, the evaluation of the system in the Ho Municipality could not be done in isolation; hence, how the reports are handled at the regional and national levels was assessed. As a result, newsletters and publications were reviewed to ascertain whether any risks were identified and what actions were implemented to reduce or mitigate such risks.

#### Assessing ADRSS attributes

The simplicity was assessed by considering the comprehension of the case definitions by the users of the system to the lowest level officer. Stakeholders’ comprehension of the reporting pathway in relation to the ADRSS flow chart was evaluated. The volume of information captured on case report forms as well as the type of data necessary to establish the case was also evaluated. Time spent collecting data, the amount of follow-up that is necessary to update data on the case, method of managing the data, including how inexpensive it is in maintaining the system was assessed.

The surveillance system’s flexibility was assessed to see how it had previously adjusted to changes. We evaluated how well the ADRSS has been well integrated with other surveillance systems, as well as how quickly the system was able to adapt to the change.

Data quality was assessed by reviewing records for completeness of case forms. Data in the database was compared to the case forms. Also, an audit trail of the electronic system was assessed to track modifications to entered data. The various levels of data validation checks were assessed.

To assess acceptability, the frequency of reports sent from the facility to the Municipal Health Directorate or to the National Pharmacovigilance Centre, report form completion rates, and timeliness of data reporting were checked by reviewing records, and computing corresponding rates. Efforts made to promote acceptability were also considered in the assessment of acceptability.

We assessed sensitivity by comparing the number of cases detected by the ADR surveillance system to the number of cases to be detected per the WHO target of 200 ADR reports per million population per year. Sensitivity was also assessed by looking at the number of signals detected by the surveillance system.

Predictive value positive was assessed using the number of case investigations completed and the proportion of confirmed ADRs (classified as “certain”) after causality assessment. The causality assessment scale was recorded.

Representativeness was assessed using the characteristics of the population under surveillance–age, sex, geographic location, healthcare facility reporting. Also, the type of medicines reported on was assessed.

Timeliness was assessed by considering the time interval between any two steps in the surveillance systems; whether the timelines were met. Timeliness of report submission, timeliness of investigation and causality assessment as well as timeliness of feedback was assessed.

Stability was assessed by considering the backlog of data not entered into the database, the sustainability of the system when Institutional Contact Persons (ICPs) were changed, unscheduled outages and downtimes for the system’s computer and electronic resources, back-up of data, and the actual amount of time required for the system to release meaningful data.

#### Assessing the usefulness of the ADRSS

The usefulness was assessed by reviewing the objectives of the system and its effect on public health interventions–policy decisions that have been implemented to promote the safe use of drugs. Also, records and interviews were done to find out whether the system detected ADRs and trends throughout the period of interest.

### Data analysis

Quantitative data were entered into Microsoft Excel^TM^ version 2019 and analyzed. Frequencies and proportions were computed. Descriptive analyses were performed on the data according to person, place and time.

### Permission and approval

Evaluation of surveillance systems is an institutionalized requirement of the Ministry of Health [19]. This evaluation exercise meets this requirement and hence needed no ethical clearance. An official request for data access was sent to the FDA and approval was granted for the evaluation to be done. Permission was also sought from the Volta Regional Director of Health Services and the Ho Municipal Director of Health Services to assess the surveillance system and for the use of data. The purpose of the project was explained to participants and they consented before interviews were done. Data obtained was stored on a computer and password protected. The data was used only for the evaluation.

## Results

### Description of interviewees

Fifty-two people were interviewed in selected facilities including 4 hospitals (high priority), 3 Health Centres (medium priority), 4 CHPS (low priority), 2 community pharmacies, the municipal Public Health Directorate, the Regional Health Directorate, and the FDA Offices at the regional and national levels. Females constituted 59.6% (31/52) of those engaged and nurses formed the highest professional group interviewed (34.6% (18/52)). Table 1 shows the distribution of interviewees by facility, sex and profession.

**Table 1:** Distribution of interviewees by facility, sex and profession.

Variables (N = 52)	Frequency (n)	Percentage (%)
**Facility**		
CHAG	2	3.9
Regional Health Directorate	2	3.9
Community Pharmacy	3	5.8
Regional FDA Office	3	5.8
CHPS	6	11.5
Health Centre	8	15.4
National Pharmacovigilance Centre	8	15.4
Government Hospital	9	17.3
Municipal Health Directorate	11	21.2
**Sex**		
Female	31	40.4
Male	21	59.6
**Profession**		
Dispensing Officers	2	3.9
Medical Officers	3	5.8
Pharmacist	5	9.6
Regulatory Officers	11	21.2
Disease Surveillance Officers	13	25
Nurse	18	34.6

### ADRSS in Ghana

**Fig 1:**
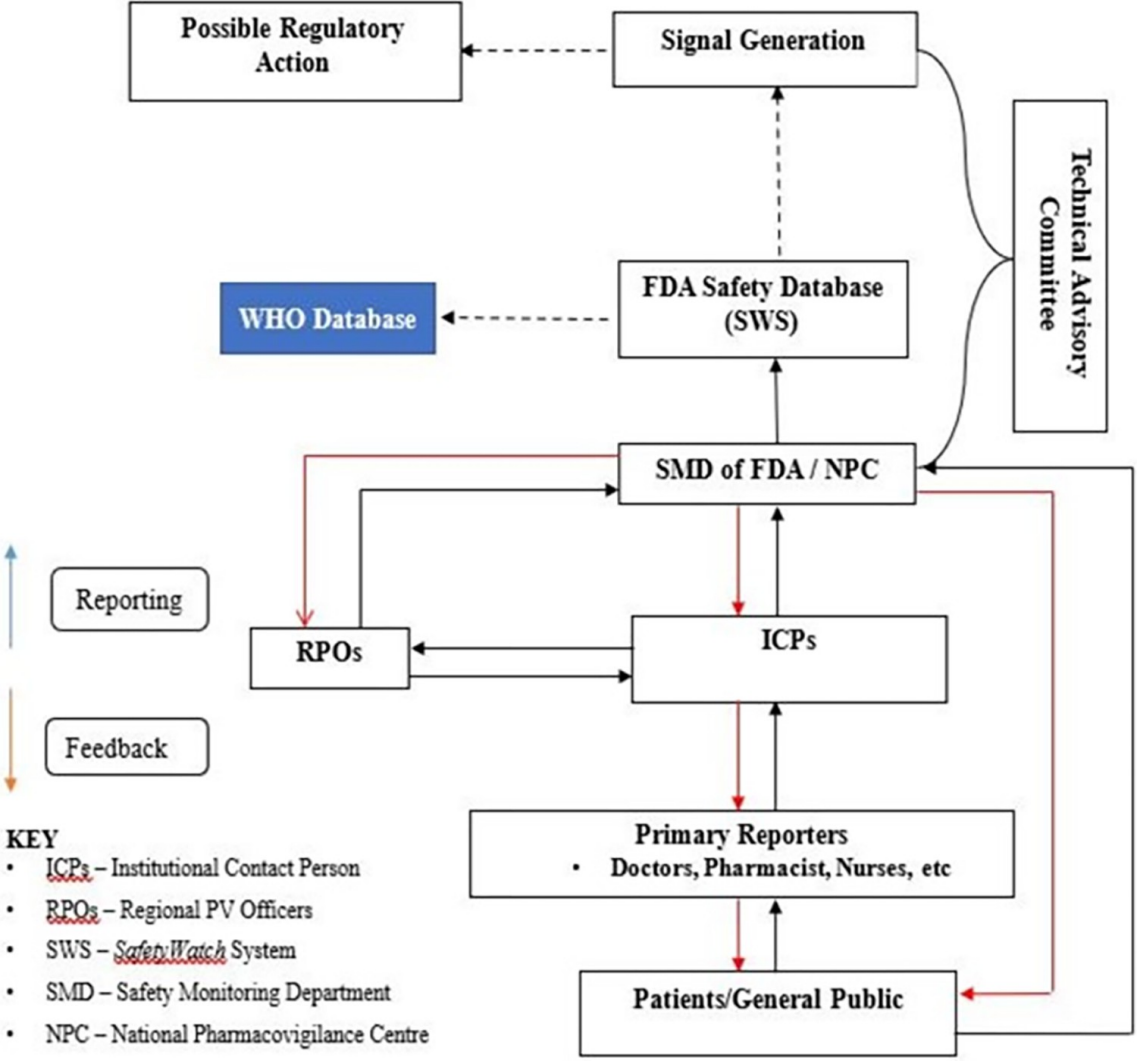
National data and information flow chart of the ADR Surveillance System used by Ho Municipality.

The system depends on passive reporting of ADRs by all healthcare professionals. Fig 1 shows the data and information flow chart of the ADRSS system in the Ho Municipality. The blue arrows indicate the reporting pathway whiles the red arrows represent the feedback pathway to reporters. Patients report to healthcare professionals (primary reporters) of any untoward experience after taking a medicine; the details of the experience are verified and recorded on the reporting form (BlueForm®) as suspected ADR. The report is sent to the Institutional Contact Persons (ICPs) who reports either to the Regional Pharmacovigilance Officer (RPO) at the FDA office in the region or the NPC (FDA Head office). The report is entered into a database (*SafetyWatch System*) that allows for analysis of the data. The system also allows for the patient or the general public to send ADR reports directly to the NPC without going through the ICPs and the RPO. Patients can report directly by using the BlueForm® or the Med Safety App or report by sending SMS to the shortcode 4015. Healthcare professionals may also report online through the *SafetyWatch* system available at http://adr.fdaghana.gov.gh/. Reports on substandard medicines, product quality, therapeutic ineffectiveness, and substandard as well as falsely-labelled medical products, are referred to the post-market surveillance team at the FDA for further investigation and possible regulatory action. The reports are subsequently processed for causality assessment (whether the suspected ADR was linked to the drug or not) by a group of experts in varying medical fields called the Technical Advisory Committee (TAC) [18, 25]. Feedback on causality assessment is sent through the same channel of reporting to the primary reporter and the patient or general public. The flowchart illustrating the reporting pathway includes broken lines to indicate potential outcomes that may occur but are not guaranteed. The ADR reports are submitted to a global pharmacovigilance database (VigiBase), which enables access to larger datasets for data mining and signal generation by other countries. Regulatory actions are taken as a result of information obtained from the ADR reports to ensure patient safety and promote rational use of medicines by the public. These actions included "Dear Healthcare Professional Letters," post-marketing studies, product label changes, changes in indications, changes in distribution categories, product withdrawal, and patient education, among others. Fig 1 indicates the Data and Information Flow Chart of the ADR Surveillance System in the Ho Municipality.

### Reporting of ADRs

**Fig 2:**
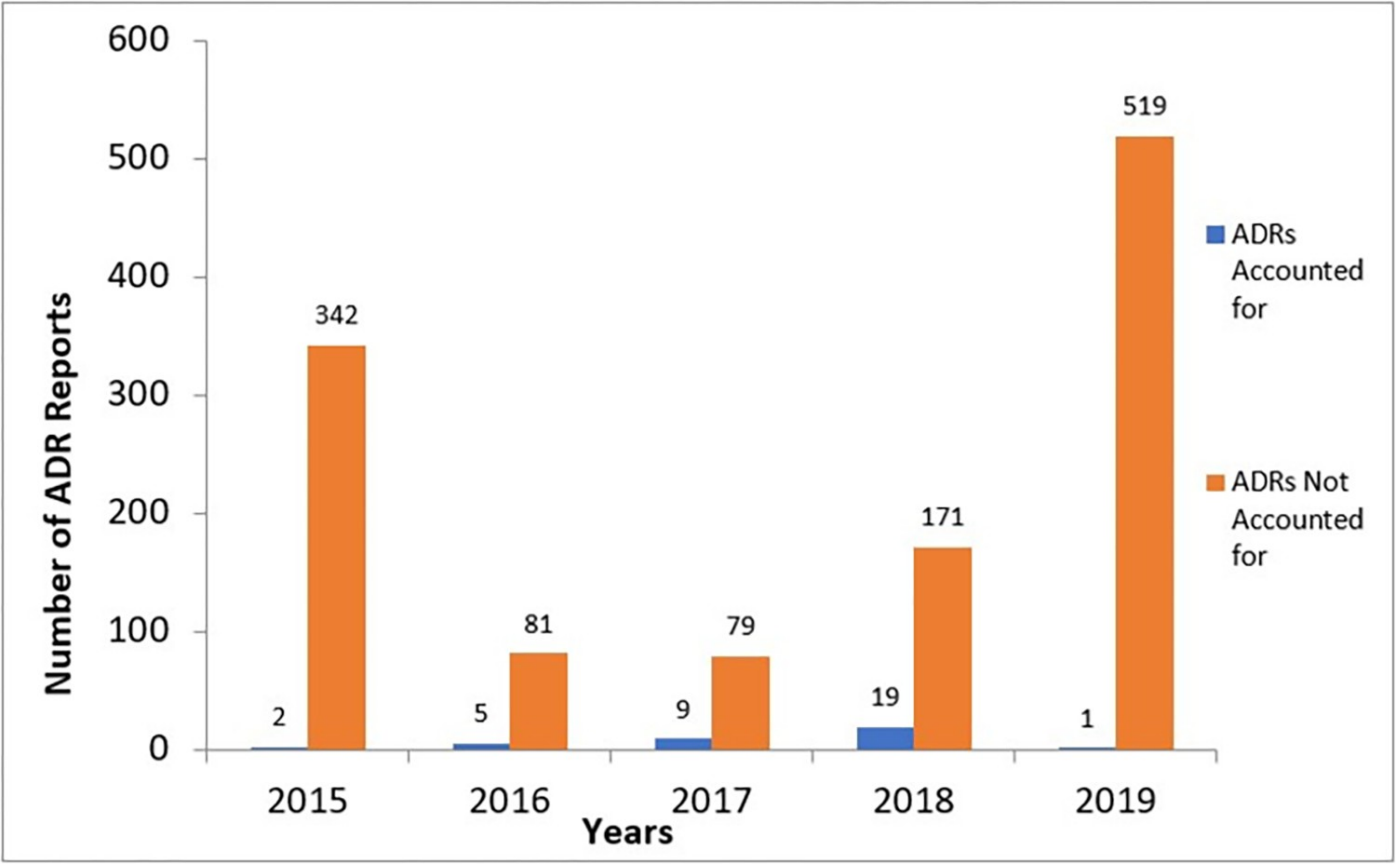
ADRs reported to NPC per year vs those not reported to NPC.

Up to 97.2% (1,192/1,227) of suspected ADRs that occurred between 2015 and 2019 were not reported to the NPC. Apart from 4 reports that were found at the health facility level, all 1,188 reports had been reported to the MHD. These reports were not forwarded to the NPC; hence, were unaccounted for at the national level. Fig 2 shows the number of ADRs reported to the NPC per year and reports that were not accounted for during the 5 years. The 1,188 reports came from Public Health Programs (PHPs) such as School Deworming Program, the Girls Iron-Folate Tablet Supplementation (GIFTS) Programme and Ivermectin Mass Drug Administration.

**Fig 3:**
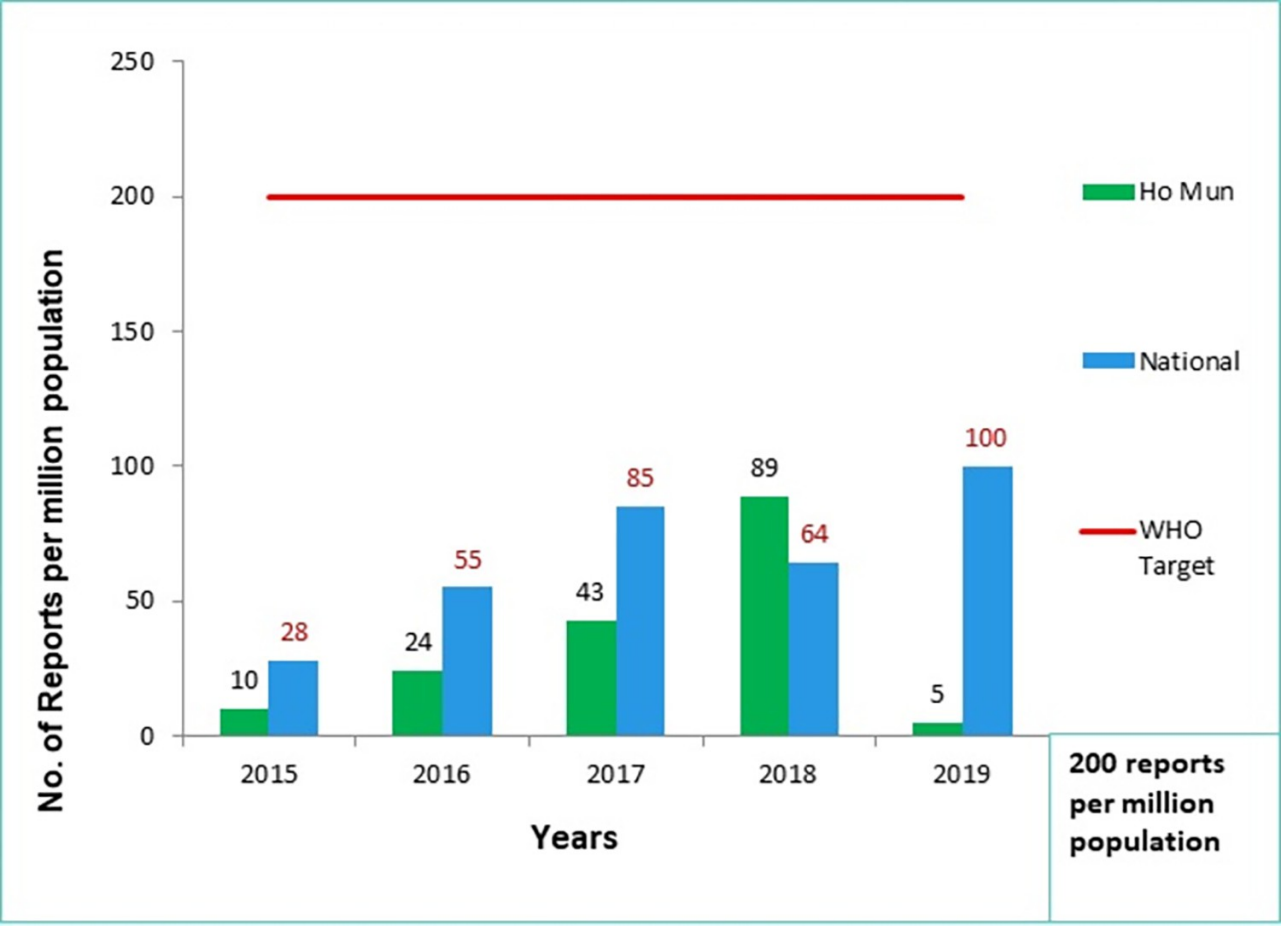
Reporting target from 2015–2019, Ho Municipality vs. National data.

**Fig 4:**
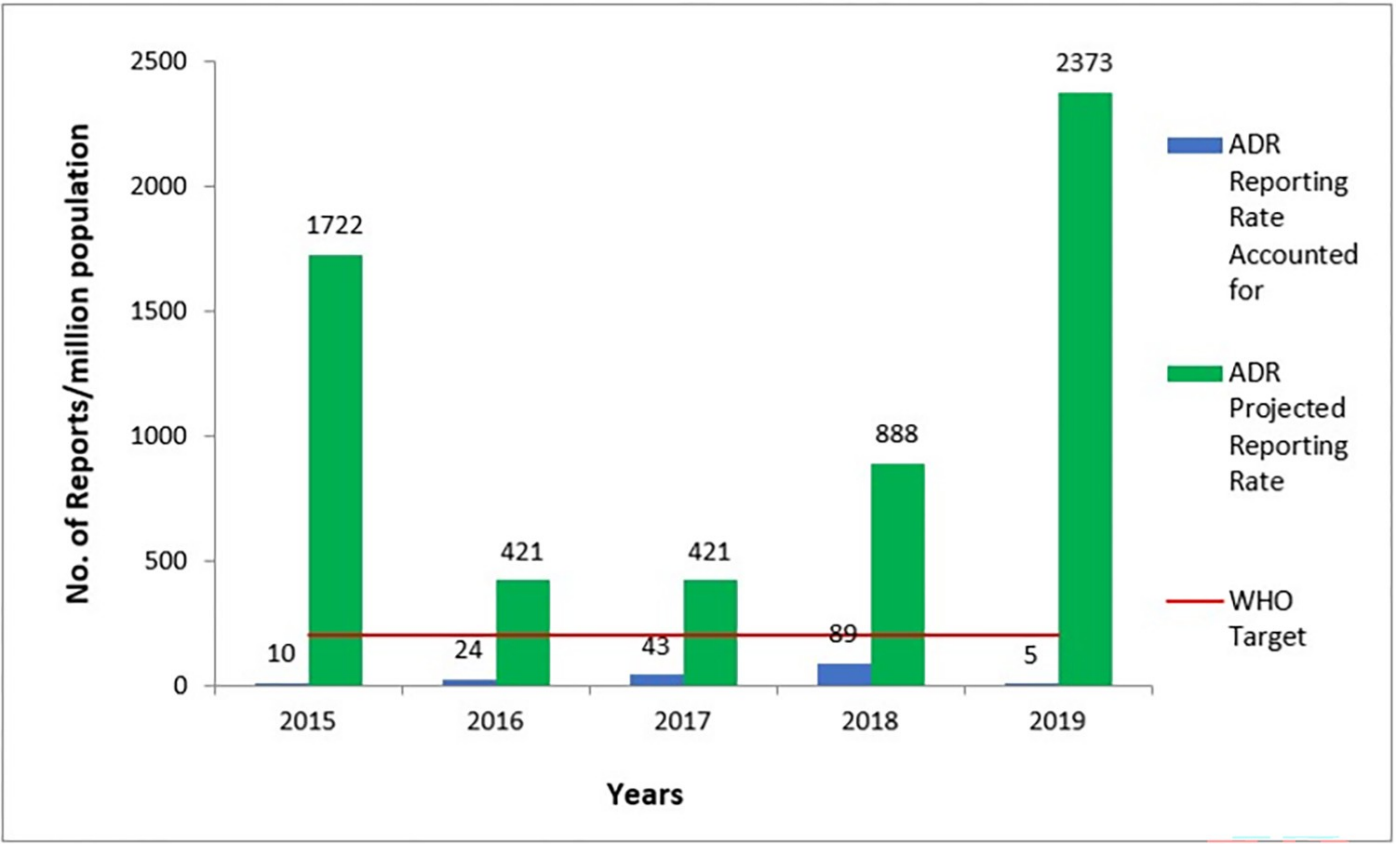
Reporting target from 2015–2019, Ho Municipality: Accounted vs unaccounted.

Fig 3 shows the ADR reporting rate per million population for both Ho Municipal and the national data for the period of evaluation. The highest for Ho municipal was 89 reports per million in the Ho Municipality in 2018 and this was more than the national rate of 64 per million population. The national projected population size in 2015 was 27,849,205; in 2016 was 28,481,945; in 2017 was 29,121,465; in 2018 was 29,767,102; and in 2019 was 30,417,856 [23]. Fig 4 also shows the projected reporting rate per Ho Municipality population if all the ADR reports were sent to the NPC and accounted for; the target would have been met for all the years within the period of evaluation.

### Meeting the ADRSS objectives

All regulatory officers engaged were aware of the case definition for an ADR and the reporting pathway. Health workers who were aware of the case definition for ADR constituted 90.2% (37/41) and 43.9% (18/41) were aware of the ADRSS and the reporting pathway. Those who could mention at least one objective of the ADRSS were 34.2% (14/41) and few, 12.2% (5/41) knew the timelines for reporting ADR. All health facilities visited did not have the flow chart of the ADRSS. There were also no ADR posters sighted at the facilities visited during the evaluation.

**Fig 5:**
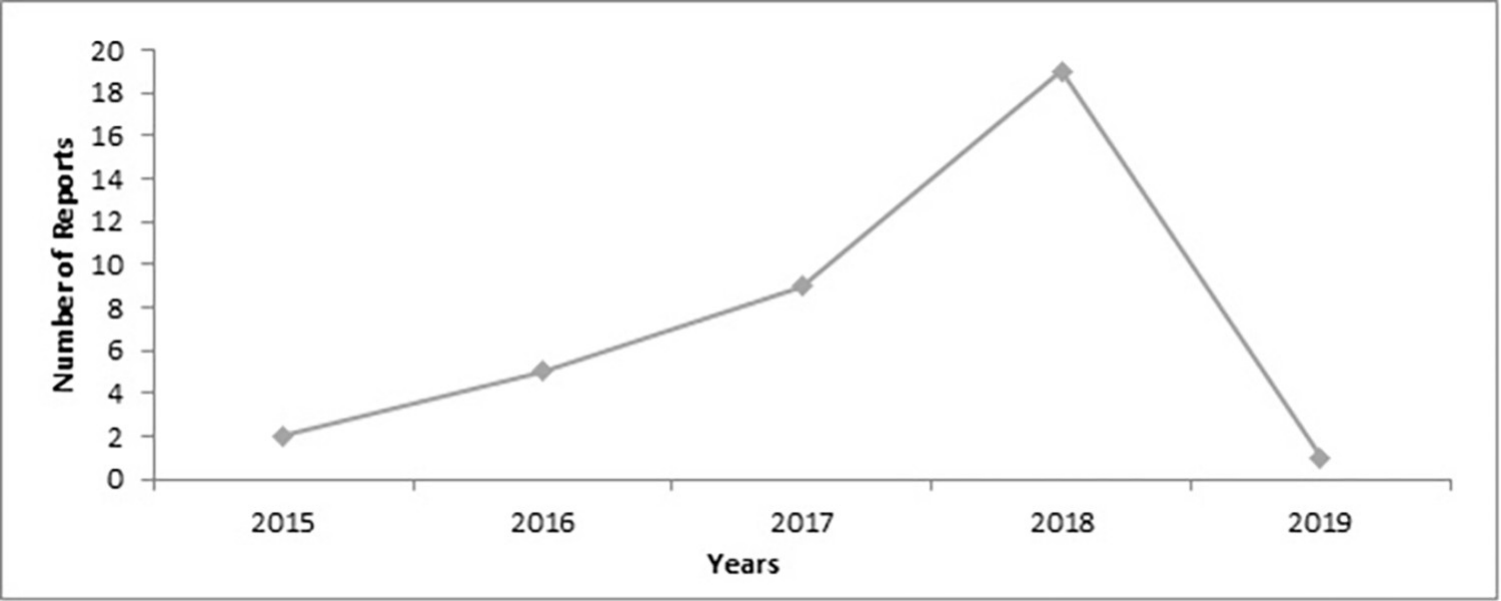
Ho Municipality ADR reporting trend, 2015–2019.

Data from the reports were entered into the *SafetyWatch System* and ADR trends were monitored at the NPC level. The RPO had a database of ADR reports in Microsoft Excel, however, charts were not drawn to monitor the trends. The MHD had charts to monitor other public health events but did not have anything on ADRs. Like the national trend, there was an increase in reporting ADRs from 2015 to 2018 in Ho Municipality; however, there was a dip in reporting in 2019 (Fig 5). At the level of the MHD, there was no existing committee for causality assessment.

**Fig 6:**
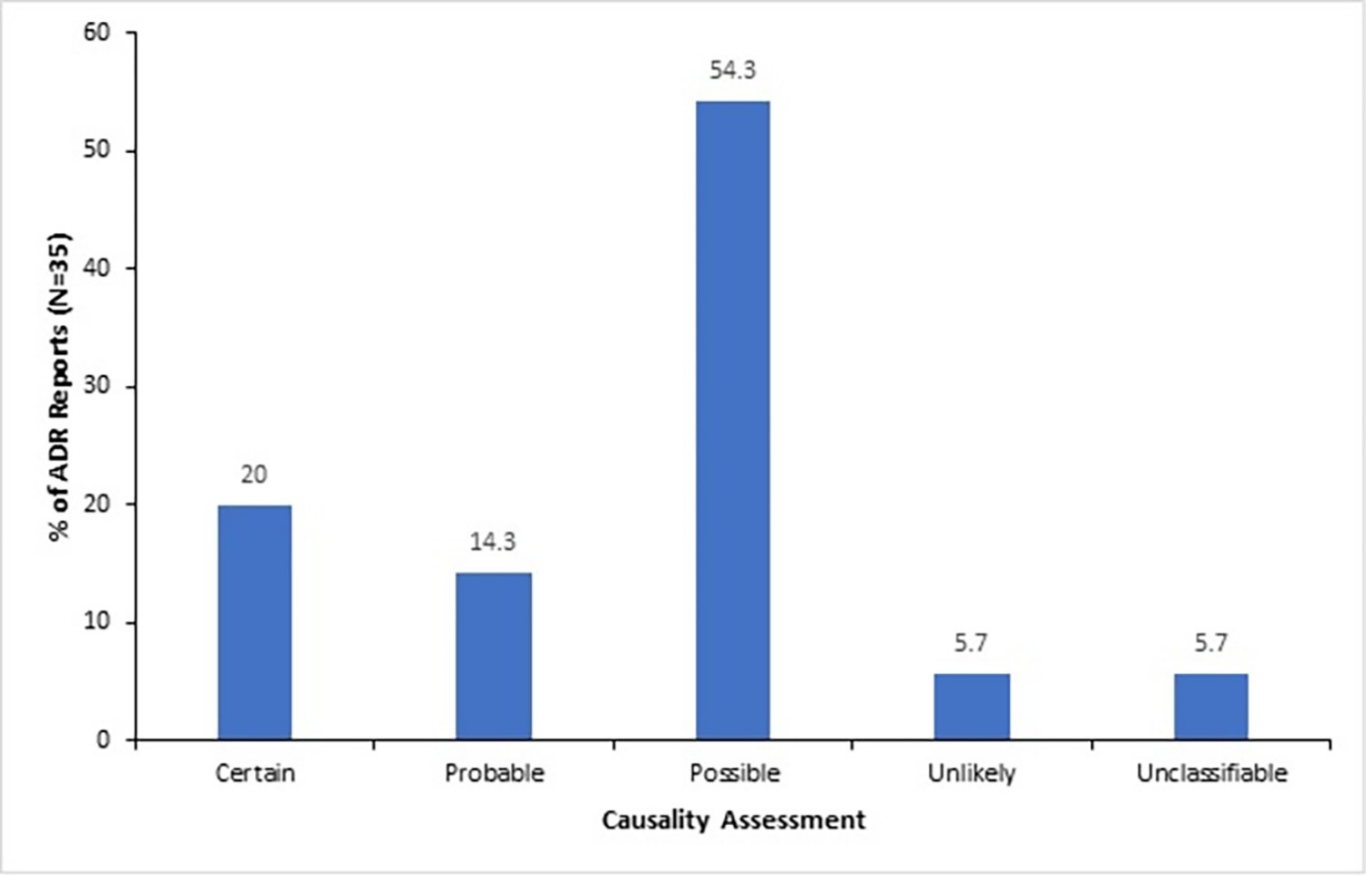
Causality assessment of ADR reports from the Ho Municipality, 2015–2019.

There were 36 reports sent to the NPC; 35 were ADR reports and 1 product quality. All the 35 ADR reports were assessed for causality as indicated in Fig 6 with the majority (54.3%) graded as ‘Possible’.

While the reports from the Ho Municipality did not have a direct influence on the risk of ADR concerns that were discovered throughout the reporting period, when combined with other information, a signal was formed about the widespread abuse of cough syrups containing codeine. Major side effects of codeine use such as bloating, blurred vision, chills, confusion, darkened urine, shortness of breath, difficulty in breathing, weak pulse, the feeling of warmth, fever, indigestion, and loss of appetite had been reported. Nationwide investigations were conducted and the signal was confirmed, leading to a policy change. There was also the risk of severe liver injury, adrenal gland problems, and harmful drug interactions associated with oral ketoconazole products. The FDA withdrew marketing authorization for Codeine-containing cough syrups and oral ketoconazole products [15]. Codeine-containing cough syrup was added to the essential medicines list when the national standard treatment guidelines were reviewed [26]. The Minister of Health, Ghana, eventually issued an instruction through Executive Instrument 167 for the restriction of importation, manufacturer and registration of codeine-containing cough syrups [27]. All stakeholders were informed.

### Attributes of the ADRSS

*Simplicity*. All regulators involved in ADRSS were aware of the reporting system; 90.2% (37/41) of the health workers were also aware and thought the system was simple. None of the facilities visited had the ADR case definitions posted publicly, even though, all had case definitions for other public health events. Interviewees found the flowchart for data and information flow (Fig 1) to be comprehensible and simple; however, there was no flowchart available in all the health facilities visited and at the Municipal Health Directorate (MHD). The national and regional offices had the flowcharts. Those interviewed did not find data required for a suspected case voluminous; information for the required 4 sections of the case report form conforms with Council for International Organization of Medical Sciences (CIOMS 1) form [28] and was easily accessible. The sections include identifiable source, patient identification, a suspect drug, and a suspect reaction. It took an average of 5 minutes to complete a form by a reporter and averagely 20 minutes for the Pharmacovigilance Officer to do follow-up and process to NPC.

*Flexibility*. There was an electronic reporting in addition to the already existing paper based. However, all the primary reporters were still using paper instead of electronic means at the time of evaluation. Not much is needed to train ICPs and users on the system update. The ADRSS was not well integrated into the other surveillance systems being used by the GHS.

*Acceptability*. All FDA & health facility staff were willing to use the system. Booklets of the ADR reporting forms were available to the hospitals classified as a high priority, however, some of the CHPS and the HCs had one copy each to show. Of the seventeen facilities visited including the FDA offices, 76.5% (13/17) already had either one or more ADR reporting forms. Reports sent from the reporter to the NPC in time (28 days) constituted 37.5% (9/24) of the total. The reports took a median time of 41 (IQR = 25, 81) calendar days from reporter to NPC. Five (5) out of the 39 healthcare professionals interviewed had ever reported on ADR and they mentioned that they all received feedback.

*Representativeness*. Reports were captured for both males and females; 53% were females. Up to 88.9% (32/36) were drug-related, covering a wide range of groups of medications. About 8.3% (3/36) of the reports were Blood & Blood products and Product Quality issues (2.8% (1/36)). Anti-tuberculosis agents (40.6% (13/32)) and other antibiotics (18.8% (6/32)) constituted the majority. Reporters were from different health professional backgrounds–Pharmacists (16.7% (6/36)), Nurses (47.2% (17/36)), Medical Doctors (13.9% (5/36)), Physician Assistant (13.9% (5/36)) & Patients (2.8% (1/36)).

In terms of representation by facilities, all the ADRs were reported by 4 out of the 35 health facilities. The 4 facilities were among the 11 classified as “high priority”. No reports were captured from private facilities.

*Timeliness*. The median time of ADR report from primary reporter to NPC was 41 (IQR = 25, 81) calendar days. Reports submitted within the timelines of 28 days constituted 37.5% (9/24). There were no timelines for processing reports to TAC but the median time was 203 (IQR = 63, 292) calendar days. Processing reports to the WHO database had no standard timelines but it took a median time of 367 (IQR = 185, 735) calendar days for the reports that had been committed. Only 20.0% (7/35) of the total reports had been committed to the WHO database. No specific timelines for feedback, however, data received indicated that feedback had been done for all reports. Dates were not available for computation.

*Data quality*. Fully completed ADR reporting forms constituted 77.1% (27/35). All completed parameters on the paper forms were consistent with the electronic data (100%). For some facilities, they had safety committees to validate the reports before sending them to the NPC. Validations were also done by the FDA regional office and the NPC. Because of the contact address section on the report form, additional information was easily sought when needed; hence, the gaps on the other 8 reports were eventually completed.

*Stability*. The regional office had a desk officer with documented tasks and targets that were appraised periodically. As part of the regional desk officer’s task, he was to promote surveillance activities by organizing workshops in the health facilities within the region. There was documented evidence of increased sensitization activities in the region within the last 5 years; however, there was a dip in sensitization activities in 2019. Not all facilities had ICPs to advance ADR surveillance activities; hence, other health workers did not own the system. Also, during the facility visits, it was observed that some nominated ICPs were no more at post (either transferred from the facility or changed job) and had to be replaced.

Computers, internet and servers were available and in good working condition at the NPC during the research period; however, the data storage system crashed in 2015 and data was lost. Measures (back-up servers) had been put in place to forestall future occurrences. The main source of funding was by the FDA, however, some activities (developing and launching of other reporting channels e.g., mobile app) were supported by grants from non-governmental agencies.

*Sensitivity*. The ADR surveillance system detected 36 cases including 1 product quality issue over the 5 years. Two cases were detected in 2015, 5 in 2016, 9 in 2017, 19 in 2018, and 1 in 2019. When expressed per 1 million population, there were 10 reports for 2015; 24 reports for 2016; 43 reports for 2017; 89 reports for 2018; and 5 reports for 2019. None of the reports sent to the NPC between 2015 and 2019 contributed directly to the signal generated by the system on the widespread abuse of codeine-containing syrup. However, Ho Municipality was part of the nation-wide investigation that resulted in regulatory actions.

*Predictive Value Positive (PVP)*. Over the 5 years, the PVP of the ADR surveillance system was calculated to be 20% (7/35). This means 20% of all reported cases were actually true ADRs. Although this suggests a high rate of false positives, it is important to acknowledge the significant impact of ADRs on healthcare costs and hospital admissions [1–6] Therefore, it is crucial to continually evaluate and enhance the system’s performance to accurately identify ADRs and reduce false positives.

Causality assessments for the reports were done by an independent group of experts at the national level using WHO-UMC System for Standardized Case Causality Assessment Scale [29]. The WHO-UMC causality assessment method considers clinical-pharmacological aspects and quality of documentation in assessing case reports for adverse drug reactions. It prioritizes the detection of unknown and unexpected reactions and uses four criteria to categorize causal association into six categories, with additional categories for unclassified or inaccessible reports.

The method can also be used to assess drug-drug interactions [30]. It is noteworthy that all suspected ADRs, regardless of causality assessment classification, are stored in an electronic database for future data mining and signal generation. This underscores the system’s commitment to improving patient safety by enabling ongoing analysis and refinement.

### Usefulness of the ADR Surveillance System

The ADRSS was able to detect and report cases in the Municipality. Trends were detected over the 5 years. The cases reported contributed to decision making during the period. The reports form part of those submitted to the WHO database, which enables all countries that have signed up to mine data and make informed decisions. The system was useful.

## Discussion

Our evaluation of the Adverse Drug Reactions Surveillance System in the Ho Municipality revealed under-reporting from 2015 to 2019. Health facilities reported only 3% to the NPC. The WHO target was not met for the reports submitted to the NPC for the years under review. Reporting rate for the period was less than the WHO target of 200 reports per 1 million population. The highest reported rate was in the year 2018 where there were 89 reports per million which were more than the national rate of 64 per million. However, if the unaccounted for had been reported to the NPC, the target would have been met for all the years with 2019 being as many as 2370 reports per million population.

Under-reporting in this Municipality is consistent with previous studies that showed gaps in reporting rates of 79.0% among physicians and 84.0% among Pharmacists in the Greater Accra region [13, 14]. We observed a higher gap in reporting rate of 97.2%. The difference in under-reporting might be due to the different target groups that were sampled. Whiles the 2 studies targeted one healthcare professional group, we targeted the facilities that included almost all the healthcare professionals. It is also comparable to a study where the median under-reporting rate was estimated to be 94% [11].

The Public Health Programs generated the majority of the ADR reports that were not forwarded to the NPC. While the MHD organized these Public Health Programs independently of the FDA, these reports would have been duly sent if MHD personnel were well-versed in the ADRSS. Healthcare professionals were the primary reporters, however, less than half of them were fully aware of the ADRSS and its reporting channels. This indicated a lack of understanding of the surveillance system and how it operated, which is a significant factor linked to poor reporting of ADRs by health professionals, according to Khan et al., 2013 [31].

All suspected ADRs received were subjected to causality assessments using the WHO-UMC scale [29], and reporters were given feedback. The method can also be used to assess drug-drug interactions [30]. It is noteworthy that all suspected ADRs, regardless of causality assessment classification, are stored in an electronic database for future data mining and signal generation. This underscores the system’s commitment to improving patient safety by enabling ongoing analysis and refinement.

However, the subjectivity of the assessments can vary due to differences in the knowledge and experience of the assessors [32]. To improve the safety and effectiveness of medications, it is recommended to involve a variety of professionals in the causality assessment and management process [33]. It was observed in this study that, the causality assessments were conducted by a group of experts from various specialties in the medical field, which lends credibility to the results. It is important to note that the quality of the assessments is reliant on the quality of available information.

The annual rise in reporting from 2015 to 2018 may be ascribed to the promotion of ADR surveillance among health personnel in the Municipality, especially in 2017 and 2018.

The system was fairly stable because it was simple. According to Muringazuva, et al., 2017, it is easier to stabilize a simple system [34]. Its stability was also due to the availability of adequate resources. There was a fully-functioning Department in the FDA responsible for ADR surveillance activities.

The information required to complete the reporting form was easily accessible and not voluminous. However, ADR reporting had not been well integrated into the other surveillance systems used by the Ghana Health Service. Since health personnel were not required to report ADRs, this might have been a contributing factor in the under-reporting of such events.

Reports had adequate sex representation; a finding consistent with other studies [35]. Females were more likely to report ADRs as indicated also by other studies [36, 37]. Antimicrobials were the most frequently implicated class of drugs during the 5 years, which is consistent with Aung et al., 2018 [38]. A similar result was found by Angamo et al., 2016 [7]. However, the target populations for these studies [7, 38] were hospitalized patients. Private facility representation in reporting ADRs, as well as community pharmacies and traditional medical centres, must improve.

Primary reporting of ADRs was inadequate and short of standard in terms of meeting reporting timelines. Late reporting makes the health system lose the opportunity to make interventions. It took a considerable time for these reports to be entered into the WHO database on average. Good quality reporting improves the possibility for signal generation, allowing appropriate regulatory measures.

The usefulness of a system depended on its attributes, and from the findings, the positives outweigh the negatives; hence can be said that the system is substantially useful even though there are areas that need improvement.

The ADR surveillance system in the Ho Municipality was partially meeting its objectives. The system was simple, flexible, stable, acceptable, representative, produced good data, had a good PPV but was partially sensitive and timely. The ADRSS had good attributes and could be a basis for exploring other avenues of capturing ADRs such as during Public Health Programs.

It is, therefore, recommended that the National Pharmacovigilance Centre through the Regional Pharmacovigilance Officer collaborates with the Municipal Health Directorate on the Public Health Programmes to ensure that all ADRs are captured. Additionally, an improvement in ADR surveillance sensitization of health workers by the RPO should encourage the use of electronic reporting of ADRs to ensure timeliness.

As a public health measure, we provided ADR forms to health facilities that did not have them, and for facilities that did not have ICPs, we obtained a list for the RPO to process.

### Conclusions

We found under-reporting of ADRs in the Ho Municipality from January 2015 to December 2019. The system was simple, flexible, stable, acceptable, representative, produced good data, had a strong PVP but was partially sensitive and timely. The ADR surveillance system was found useful and partially met its objectives based on the attributes assessed.

## Supporting information

S1 Checklist (DOCX)

## List of abbreviations

ADR: Adverse Drug Reactions
ADRSS: Adverse Drug Reactions Surveillance System
CDC: Centers for Disease Control and Preventio
CHAG: Christian Health Association of Ghana
CHPS: Community-based Health Planning and Services
FDA: Food Drugs Authority
GHS: Ghana Health Service
HCP: Healthcare Professional
ICP: Institutional Contact Persons
MHD: Municipal Health Directorate
NMRA: National Medicines Regulatory agencies
NPC: National Pharmacovigilance Centre
OPD: Out-Patient Department
PVP: Predictive value positive
RPO: Regional Pharmacovigilance Officer
WHO GBT: World Health Organization Global Benchmarking Tool
WHO-UMC: World Health Organization Upsalla Monitoring Centre
