# Supplementary material for: Under-reporting of adverse drug reactions: Surveillance system evaluation in Ho Municipality of the Volta Region, Ghana
Source: PLoS One. 2023 Sep 12;18(9):e0291482. doi: 10.1371/journal.pone.0291482 (PMC10497160; doi:10.1371/journal.pone.0291482)
Supplement: S1 Checklist — (DOCX) [file pone.0291482.s001.docx]

**DEMOGRAPHICS**

Institution:

Age:

Sex:

Type of health care worker: Doctor[ ], Nurse[ ], Pharmacist[ ], Other[ ].

Rank:

Years of practice:

Department:

Unit/ Specialisation:

Ward: Medical[ ] Surgical[ ]Paediatrics[ ]Obst And Gynae[ ]Oncology[ ] Hematology[ ] Others(please indicate) …………………………………………………………

Mobile Phone number:

Email address:

**Interview (National Pharmacovigilance Centre)**

**Legal provisions for existence of a national vigilance system.**

1. Is there any legal basis to establish a surveillance system for ADRs?
2. How do you enforce the provision?
   1. Does it provide for adequate and proportional sanctions, penalties and prosecution of violations of the applicable legislation?
3. How does the legislation define the duties, powers, and responsibilities of the regulatory authority to manage risks associated with the use of medical products, including:
   1. Collection of data on the safety of the medical products
   2. Analysis and investigation of this data, and
   3. Adoption of regulatory measures by the health authority?
4. Are there Guidelines or other documentation encouraging healthcare institutions and consumers to report ADRs to the NRA?

**Guidelines for planning, conducting, monitoring, and reporting of vigilance activities.**

1. How do you go about ADR surveillance activities? Any guidelines/SOPs on planning, conducting, monitoring and reporting? The documentation should contain:
   1. Objectives of the vigilance system;
   2. List of events (ADRs) to be reported
   3. Clear definitions of terminology relevant for analysis and response (e.g., adverse reaction, medication error, coincidental error, program error, serious event, and cluster event);
   4. Information on how to report (i.e., who, how, where, when and to whom);
   5. All medical products to be included in the reporting system (i.e., reporting is not restricted to certain products or sectors);
   6. Procedures for analyzing data;
   7. Procedures for providing feedback of findings to key players (e.g. reporters of complaints, informers, parents, caregivers, associations and communities) and for relevant follow-up actions;
   8. Guidance for investigations and actions to be taken in case of serious events or a cluster of events
   9. Guidance on assessment of the balance between risks and benefits for medical products;
   10. Guidance on crisis prevention and management;
   11. Guidance on communication of vigilance information;
   12. Definition of the individuals in charge;
   13. Guidance on potential vigilance-related differences and particularities among different medical products (e.g. medical devices, herbal medicinal products, food products).
2. Are these guidelines consistent with other internationally accepted Guidelines? (WHO)

**Defined organizational structure with clear responsibilities to conduct vigilance activities**

1. How do the surveillance activities fit into the organizational structure?
   1. Organization chart along with identification of the particular structures implementing the function.
   2. Documentation clarifying roles and responsibilities of the structures implementing vigilance activities.
   3. Documentation identifying established mechanisms of coordination (e.g., committees, internal work-sharing, and workflow) among structures, if any, which take part in vigilance activities.
2. Are there any implemented and documented procedures and mechanisms that allow the involvement, coordination and communication among stakeholders?
   1. List of stakeholders relevant to vigilance of different medical product streams
   2. Agreements, MOUs and standard operating procedures (SOPs) defining the means of communication and collaboration among the identified stakeholders
   3. Example records of communication and collaboration demonstrating implementation of the above-mentioned procedures.
   4. Guidelines or SOPs defining procedures for external and internal communications.
   5. Documentation that regular meetings and other formal or official communication among above-mentioned stakeholders take place. This documentation should provide evidence for a systematic and well-established communication process.
   6. Documentation of paths of communication and reporting.
   7. Platforms for information sharing and exchange.

**Sufficient competent staff (i.e., education, training, skills and experience) assigned to perform vigilance activities**

1. How many people are involved in each of the documented activities along the entire surveillance process flow?
2. What level of education is required? Any required skills and experiences?
3. How are people trained on the job? Availability of any documentation on the list of the requisite skills and training for each position. Is there a training plan?
4. Are there documented job descriptions of staff? Training records available?

**Vigilance procedures and tools are in place and implemented for collection and assessment of ADRs**

1. What reporting system is employed and available to healthcare professionals and patients – paper-based or an electronic-based?
2. How are ADRs detected and processed?
   1. Documented procedural steps (flow chart)
3. What system is used for detection and receipt of vigilance events and ADR reports that is complemented with reporting systems (e.g., either active or passive, and either sentinel or country- or state-wide)?
   1. Is it sensitive to detect serious events or clusters of events? The number and rates of reports within defined periods, ADRs following off-label use, and a breakdown of reports that compares district or regional reporting activities for the different products and populations that are involved in vigilance reporting.
4. Are there timelines for review, analysis, and causality assessment of ADRs?
   1. How long does it take for investigations to be initiated following reporting?
   2. How are investigations done?*
   3. Are there records of ADRs and how are they maintained?
5. Are the procedures consistent with the set out objectives?
6. Are there thresholds for withdrawing or recalling products that are detected by the system?
7. Has there been a change in the case definition?
8. Has there been any change in the system and how has it affected your work?
9. How does the vigilance system work with other systems?
10. Is there evidence that the reports are analyzed on a regular basis?
11. How is data collected and analyzed? (e.g. calculation of incidence rates or the assessment of causality).
12. Is there a standing advisory committee?
    1. Terms of reference and standard procedures for the expert committee
    2. Records of discussions and decisions of the expert committee for review of serious vigilance events over the last five years.
    3. Procedures for convening the expert committee when needed to provide advice and recommendations.
    4. Procedures for follow-up and implementation of the expert committee recommendations, including procedures for re-convening the expert committee as needed to review progress.
13. Is there a national database system that is compatible with ICH E2B, to code, collate, and store data and reports and to analyze vigilance data?
14. Are reports transmitted to international collaborators’ databases along with the frequency of submission?
15. How are the following done?
    1. Assessment of risk, analysis and evaluation of vigilance data, and identification of trends
    2. Processes for signal detection
    3. Use of statistical tools to calculate reporting disproportionalities such as Proportional Reporting Ratio
    4. Initiation of appropriate actions at the national or sub-national level when needed*:
       1. Addition to national vigilance database
       2. Follow up or further analysis
       3. Referral for comprehensive investigation or systematic causality assessment
       4. Issue of safety alerts and/or batch or product recalls
       5. Other regulatory decisions including product withdrawal and/or revocation of marketing authorization.
16. How are recommendations arising from causality assessment acted upon?
17. Any records of actions taken within the last 5 years?
18. Are reports of notifications, data analyses, committee meetings, and other related records available?
19. Are there records on signals detected from national, regional or linked international databases?
20. Any back-up servers, power supplies and data storage? Challenges.

**Mechanism in place to evaluate the surveillance system output**

1. Is there a list of regulatory decisions and action taken based on ADR reports?
2. Are there specific periodic evaluations of the system and to what extent?

**Vigilance activities and relevant feedback are appropriately communicated to the public**.

1. Who are your key stakeholders?
2. Is there a risk communication plan for regular feedback to all stakeholders?
   1. Risk communication plan and procedures for communication with different stakeholders involved in the vigilance system.
   2. Examples of shared information among those stakeholders.
   3. Records of communication (e.g., social media, newsletters, websites, or publications) among the NRA and those stakeholders.
   4. Records of regular meetings among the vigilance relevant stakeholders.
3. How do you ensure regular feedback to the various levels of reporting and the patient?
   1. Proper mechanism established (SOPs, Guidelines, evidence of the feedback)
   2. Serious events/reactions and clusters of ADRs communicated appropriately to the public (including patients, parents and caregivers).
   3. How often are feedback given – systematically or ad hoc basis?
   4. How long does the feedback take to reach various levels on the average?
4. Any approaches towards management of potential risks that may contribute significantly to risk reduction or elimination?
   1. Are investigation reports of public concerns or summaries of these reports made available to the public? –
      1. Records of communications to the public community (e.g., social media, newsletters, and websites);
      2. Information bulletins and documentation of public awareness sessions and campaigns;
      3. Published alerts, assessments and investigation reports.

**Interview (ICPs, Healthworker)**

1. How is ADR detected?
2. How is ADR (blue form) processed once detected?
   1. Timeline, which method is used (paper, app or database), steps, who to report to, records, SOPs
3. Where do you find cases?
4. How were you appointed as an Institution Contact Person (ICP)?
   1. Training, workshops, incentives and their respective frequencies
5. Do you have enough adverse drug reaction reporting forms (blue form) available all the time?
6. Are there key elements that have to be completed on the reporting form?
7. How easy is it to complete the forms?
8. Why are you motivated to report ADRs?
9. Any suggestions to improve the system?

OBJECTIVES AND PURPOSE OF THE ADR SURVEILLANCE SYSTEM

|  | Objectives of the system |
| --- | --- |
|  | Ways in which objectives are met |
|  | How does the system work |

SIMPLICITY

Having an efficient structure and ease of operation of the ADR surveillance system

|  | Availability of a flow chart of the ADR surveillance system |
| --- | --- |
|  | How are cases identified |
|  | Case definition for ADR and ease of application |
|  | Is the ADR case definition easy to apply |
|  | Amount and type of other data to be collected on ADR cases (e.g., demographic, behavioral, and exposure information for the health-related event) |
|  | Who is responsible for submitting case reports |
|  | In what ways are ADR case reports transmitted (Electronic, telephone, hard-copy) |
|  | Ease of gathering and reporting case reports |
|  | Who does data analysis |
|  | Frequency of analysis |
|  | Type of analysis |
|  | Is data management computerized |
|  | Method of disseminating information |
|  | Are there any steps or procedures that the system can do away with and still be effective |
|  | Average time spent between steps and analysis of data |
|  | Number of follow ups to be made |
|  | Sample storage and transportation procedure |
|  | Method of managing the data, including time spent on transferring, entering, editing, storing, and backing up data |
|  | Time spent on system |
|  | Staff training and supervision (training requirements and level of qualification) |
|  | Information flow |
|  | Time spent on system |

ACCEPTABILITY

The willingness of those involved in the ADR surveillance to participate in the process

|  | District participation rate |
| --- | --- |
|  | Report form completion rates |
|  | Question refusal rates |
| . | Timeliness of data reporting |
|  | Completeness of report forms |
|  | Subjects refusal rates |

STABILITY

The reliability of the ADR system, and its ability to work when you need it to

|  | Is there any legal basis to establish a surveillance system for ADRs? |
| --- | --- |
|  | Are there Guidelines or other documentation encouraging healthcare institutions and consumers to report ADRs to the NRA? |
|  | What are some of the reasons why the system may stop operating |
|  | The number of unscheduled outages |
|  | Staff absenteeism or shortage |
|  | The costs of repairing the system (parts, service, and amount of time required for the repair if some parts are operated electronically) |
|  | The percentage of time the system is operating fully |
|  | The desired and actual amount of time required for the system to collect or receive data |
|  | The desired and actual amount of time required for the system to manage the data (may include transfer, entry, editing, storage and back-up) |
|  | The desired and actual amount of time required for the system to release data |
|  | Back-ups for system |

DATA QUALITY

Completeness and validity of the data in the system

|  | Identify completion percentage of ADR forms |
| --- | --- |
|  | Source data verification – national data to medical records |
|  | Clarity of hardcopy forms |
|  | Quality of training of persons to complete forms |
|  | Supervision |

TIMELINESS

The ability of the surveillance system to take appropriate action without delay between any two or more steps

|  | Time between steps of the system based on timelines |
| --- | --- |
|  | Time required for investigation to be completed based on timelines |
|  | Time required for validation of reports to national database |
|  | Time required for decision-making based on timelines |
|  | Time required for feedback based on timelines |
|  | Time required for regulatory actions |
|  | Factors that could delay in of the above parameters |

FLEXIBILITY

The property of being able to adapt to changing needs and conditions

|  | Have there been any changes made to the system |
| --- | --- |
|  | Components modified (e.g. case definition, paper to electronic etc) |
|  | Cost of changes – time, energy, expertise, load |
|  | Amount of training required to match up change |

REPRESENTATIVENESS

The property of accurately describing where and in whom the health event of interest is occurring

|  | Availability of ICPs in facilities and those who report |
| --- | --- |
|  | Characteristics of population under surveillance (to age, sex, geographic location, socioeconomic status and access to health care) |
|  | Characteristics of population cases are identified from (person, place and time) |

SENSITIVITY

How well a surveillance system captures the ADR

|  | How many cases are to be detected per period (National & WHO guidelines) |
| --- | --- |
|  | Cases detected |
|  | Number of clusters detected |
|  | Visit a health facility and review records of ADRs to find out how many cases were documented for the past year |
|  | Visit DHMT to review documents to identify how many suspected cases were reported |

PREDICTIVE VALUE POSITIVE

The proportion of persons identified as cases in the surveillance system who truly have the health event.

|  | Total number of confirmed ADRs after causality assessment per Total number of reported ADR cases expressed in per million population |
| --- | --- |
